# A Few Thoughts on Professional Qualifications, for Dental Tyros

**Published:** 1856-07

**Authors:** 


### ARTICLE III.

### *A few Thoughts on Professional Qualifications, for Dental Tyros.*

WE have no fear of unintentionally offending the youth of our profession by the use of the word in our caption, as they can at once refer to various lexicographers and ascertain that tyro means one not yet master of his art. The explanation may not be altogether unwise, as we know of high dudgeon having been taken by a few gawky students, who were charitably termed "*novices*" and "*new travelers*" on the road to professional excellence, when lo! and behold, they became restive and turbulent, from the force of habit, we presume, as nothing could appease their outraged dignity—but a truce to trifles, let us turn to grave points.

Young men generally enter our profession with but two prevailing ideas; the first money making, the second, an easy luxurious occupation. True, many feel that their position is greatly advanced, and no doubt is, by having themselves nominally enrolled as a member of a profession which lays claim to high rank and noble attainment, and they consequently look upon any change in this particular as a happy hit, if the public but yield in any degree to their claims.

Again, some engage in the pursuit without such craft, but entertaining a mere respect for a calling which they imagine easily acquired, easily fulfilled and entirely divested of the labor of mechanical trades; feeling confident that it will most of all gloriously enrich, they turn to it, without those mental acquirements demanded by law and medicine—without the capital needed in commercial and mercantile pursuits, looking upon dentistry as an occupation freed from these difficulties, giving ease, profit and renown to all its members, however variously possessed of ability. But most strange to say, this impression exists up to actual experience, when they discover the unimagined intricacies of practice and the immense advantage of a liberal education. The power and effect which cultivated tastes and personal elegance command, the polish of manner and high breeding required, the patient forbearance necessary in the intercourse with erratic character, nervous irritability and misguided conduct so often met with in the numerously attended office—the enduring energy demanded in mastering the personal peculiarities on the one hand, and the physical obstacles on the other; the unceasing industry necessary to perfect any chemical or mechanical process—the indispensable absence of all discouragement—the years consumed in combating unknown difficulties and the constant application of the mind necessary to comprehend the laws presiding over the different processes in dental mechanism and the secrets of animal economy.

The intense labor, fatigue and unavoidable discomfiture at times operate with fearful impression and make the stoutest heart tremble, and yet, oh! glorious confidence, we find the plough deserted for this life-absorbing pursuit, and the hammer and chisel lying unused for the luxury of dental practice. The broken down artizan embraces this profession to heal the wounds his pride has suffered and to fill his empty pockets, and that too in the most incredibly short time. After a few months passed in pouring over ponderous books, bound in calf skin, and filled with Greek and Hebrew, they come forth fully prepared for the combat, and ready to confront the oldest and best of the profession. Some, because they can push a needle through a camel's eye, feel themselves peculiarly drawn to the profession and capable of fulfilling its entire want, forgetful or ignorant of the fact that it is necessary to swallow a few such monsters before becoming a dentist.

One gentleman founded his claim of suitability upon the fact, of having, whilst duck shooting, forced a number two shot into a carious tooth, which preserving it, secured to the profession a great man, although he may be somewhat doubtful as to the grammatical composition of a simple sentence; yet he says he can "fill a tooth as well as any man and insert whole sets of artificial ones."

A certain barber declares he was born a good dentist, from his having two teeth at birth, and always has pulled teeth because it came natural to him, and made him money without trouble. This list we could increase ad infinitum, but as they are anything but pleasant reminiscences, we will proceed by reminding the uninitiated of the importance of fully comprehending the labor that is necessary in acquiring a competent knowledge which will enable him to strike at the highest excellence; the keeping of this secret, in the mind's eye, will enable one to appreciate the vast amount of study which dental practice involves. The whole field of medicine becomes a leading study, so as to determine what effects are likely to be produced in the mouth, not only as a sequallæ of the various diseases, but as a consequence of the kinds of treatment employed, and the peculiar effect, whether local or general, of remedies administered; for we scarcely know of a disease or its treatment, that, under some form or circumstance, does not involve the mouth or connecting relationship. This knowledge may not generally be found indispensable, but the want of such knowledge frequently occurs and its possession offers supreme advantage, not only in the skillful treatment of such cases, but in the estimation given to its possessor by the public, tending to increase and enlarge his sphere of usefulness and his pecuniary emolument.

Then, again, dental mechanics, in all its bearings, involve the application of chemistry, to enable the artist to make the several ingredients he employs, to purify his composition, to reduce his various materials to convertible forms, in order to supply his wants, to make improvements upon, or to add to those processes already adopted, to analyse substances and to illiminate therefrom useful and obnoxious particles of matter.

Metallurgy and its vast field of useful facts in the combination of metals, and the study of their as yet undefined law of union, their varying degrees of liquefaction alone and in combination, their density, oxydation, galvanic action, gravity, durability, ductility, maleability, &c., offers almost an unexplored region, demanding the highest capacity and energy in the study of it, and, here let me say, a lifetime could be spent and still leave something for successors to learn in the working of metals, in connection with the dental art.

Mechanical dentistry has derived the greatest advantage during a few years by its incorporation with the art of porcelain manufacture, as through it the most beautiful and artistic results have been produced, rendering a knowledge of this process indispensable, so as to compete with the possessor of this superiority, as well as to improve upon present perfections. Here let me insist upon the necessity of a thorough knowledge of this most intricate and difficult art, whose vicissitudes and almost unaccountable changes, render its highest present perfection a somewhat unsatisfactory and most disheartening study, still leaving for future science and labor the amplification of this branch of dentistry. Even as it is at present perfected, it requires a great amount of experience and labor, presenting anything but the promise of ease.

It is somewhat curious to observe the astonishment of both patient and pupil when first witnessing the necessary physical labor bestowed in filling a delicate tooth, even of the smallest size. Their ideas of the practice generally resemble a midnight view of flowers or the common interpretation of genius, whose labors are considered the result of heaven bestowed favors, not of human powers and toil-worn applications.

It is not surprising then that dental ligaments are occasionally found whose existence confers the secret of pain in tooth drawing, or that young operators frequently discover some system about to revolutionize dental practice, when, alas, a short time proves their scheme to be an abandoned one of some departed progenitor of the science.

Our art can, in no degree, be conferred by the laying on of hands, of the most gifted; the peculiar ability to operate comes not thus; no combination of mechanism has as yet been found able to suit the varying forms of mechanical dentures; no learned lectures on temperament and physiognomy have as yet been able to make the dental student understand the restoration of natural expression, or to lay down laws whose general application will result in the re-establishment of comparative youth, comfort and beauty, and yet with pride and gratitude do we frequently see such results being conferred on patients by the labor and industry of men of talent and long experience in our profession.

Such superiority is reached only through the toil of the wisely applied industry of years, and cannot be imparted by oral instruction, but is delved out in fatigue and by unwavering energy, and exists only with the possessor of these giant qualities.

We have no wish to discourage untried ability or deserving aspirants, being ever ready to help all such with our humble means, but we do denounce the encouragement of men, who, because they can *whittle a piece of wood* to any shape, feel themselves capable of dental practice—because they feel mortification in following a lowly, yet honorable occupation, suitable to them by nature and physique, should seek dentistry from an unmanly shame of the calling of their fathers, without those qualifications arising from a thorough education, seeking the enlargement of the scientific and the useful.

It is by such hybrid characters that the profession is pressed down, that the mass is degraded, and the calling a doubtful cognomen among strangers. It is these individuals who decry a branch of the profession, and attribute to it a deteriorating influence, forgetting that this very department has achieved, through the labor and toil of skillful men, a perfection that is attained in no other science or art. Eminently honorable because useful; most artistic, because it restores to nature its lost beauty; elevating, because in reaching its perfection, it embraces in its study all the influences acting upon the mind and heart developed in the countenance, whose entire restoration is in its domain and whose fundamental law is harmony. True, they do not perceive these facts, but are they any the more wanting. Surgery amputates a limb, but does not restore one. Operative dentistry prevents disease from going further but does not recover the lost form of appearance of the tooth; medicine sometimes through analogy prevents death, a point yielded only through courtesy, as positive proof must be forever wanting to establish the fact. The supply of almost all human wants is only in a measure complete, still to be improved and added to, but mechanical dentistry in the hands of honorable skill reaches a perfection beyond all. In the first place, the usefulness of some artificial teeth is as complete as the natural organs, so perfectly adapted as often to dispel from the mind of the patient, in many cases, the consciousness of their presence, so completely harmonizing with the complexion, so conformable in shape to the countenance, as to leave no point of detection, no discrepancy. The lines and rotundity almost of youth are restored, the mouth regaining its wonted expression. Its function recovered to the great advantage of the whole economy of the body, replete with beauty and usefulness.

Little men, and *sometimes* those fortunate in worldly possessions, decry this branch to the preference of the other, the first, because their conceptions of its requirements through ignorance, are absolutely at fault, being incapacitated to embrace the knowledge and means necessary to be acquired to strike at perfection, they fail repeatedly, and denounce their own errors and incapabilty in abusing a branch of the profession entirely out of their reach. The second decline its practice, because it involves labor and toil much greater than in the operation of filling teeth, because the responsibilities are incomparably greater, because the consequences of the two departments are entirely different. In the one there is a never ceasing requirement of perfection, not only from the patient, but from the community in which they reside, with a certainty of exposure and condemnation if a fault be committed, or in the result of failure. In the other, there is an analogy between its practice and the errors of an unfortunate physician who buries all his prescriptions with his patients. A good or bad filling is covered by years of entire concealment and absolute forgetfulness, and this occurs in almost all cases. The community of friends of the patient are absolutely excluded from all possible means of judging of the merits or demerits of the case, except as the grace of manner of the operator has impressed the patient, this being of necessity the only topic upon which either can decide. The one has to do with one material only and that prepared by others, whose use involves time, ranging from thirty minutes to three hours, when the fee is received and the matter forgotten by all, at least for years. The other involves the employment of the arts and sciences, in the moulding of the most numerous and intricate substances into intelligent shapes and suitable compounds, whose manipulation requires weeks and months of labor. The great trouble is found in the intense difficulties to be encountered on the one hand and the comparative simplicity on the other. In the magnitude of the responsibility, and publicity in one, the unavoidable obscurity and inobservant character of the other. The manipulations so delicate, are still made in pungent, blackening chemicals, earths, metals, gums, &c., of the laboratory, the toil and burning heat of which though resolutely endured are not, in their nature, always successful. Those of the operating room are performed without contact with materials, whose use there is any room to doubt or to discover or prepare, their easy labors are performed over a beaten course, whose result is the same one day as another, and whose pecuniary success depends in a great measure on personal accomplishment.

We do not wish to detract from the skill and dexterity required in operating, or intend to give the idea that little is required in this department; for there is abundant proof of its imperfect accomplishment in the fact, that, perhaps, half the operations performed are entire failures, the remaining half being protected by doubt and simplicity.

But there is a great difference, seemingly overlooked by the profession, to the disadvantage of mechanical dentistry. In the operative department there are peculiar difficulties of course, but these for the most part are overcome by skill and manual dexterity, whilst the other demands all of these and more, for it has not only to vanquish the obstacles to the most exact manipulations, but it has to do with laws and actions so subtile as to defy, in many cases, the longest experience, the most acute knowledge of chemistry, for defeat comes unexpectedly near the completion of a process, by the casual introduction of some unseen, yet potent agent, whose discovery is only to be made through the aid of science and enduring toil. "Every theory which urges man to labor and research, which excites acuteness and sustains perseverance, is a gain to science; for it is labor and research which lead to discoveries." So says the greatest man of modern science, and with whom every man of sense will agree, and by this law mechanical dentistry will ever hold a high position with capable and honorable men, for its perfection can only be reached through labor and research, and the application of enduring perseverance with the most intense acuteness.

